# Research progress of connexins in epileptogensis

**DOI:** 10.1186/s42494-025-00203-9

**Published:** 2025-03-15

**Authors:** Jiaqi Wang, Suhui Kuang, Zhirong Wei, Shuli Liang

**Affiliations:** 1 Department of Functional Neurosurgery, National Center for Children’s Health, Beijing Children’s Hospital, Capital Medical University, No. 56, South Lishi Road, Xicheng District, Beijing, 100045 China; 2 Key Laboratory of Major Diseases in Children, Ministry of Education, Beijing, 100045 China

**Keywords:** Connexin, Gap junction, Hemichannel, Epilepsy, Wnt pathway

## Abstract

Epilepsy, a chronic neurological disorder, is characterized by dysfunction in neural networks. Gap junctions and hemichannels, which are integral to the astrocyte connection network, play a critical role in epilepsy. Connexins, the components of astrocyte gap junctions and hemichannels, can be activated to transfer glutamate, adenosine triphosphate, and other chemicals, potentially leading to seizures. Connexins therefore hold significant potential for epilepsy treatment. This review focuses on connexin 43 and provides a brief overview of other connexins and pannexin 1. Understanding the relationship between connexins and epilepsy offers theoretical support for developing new antiseizure medications.

## Background

Epilepsy is one of the most common neurological disorders, affecting over 70 million people worldwide [1]. It significantly impacts patients’ psychosomatic health and quality of life. Although many antiseizure medications are available, 30% to 40% of patients still have seizures despite the use of two or more antiseizure medications, known as drug-resistant epilepsy [2–4]. Additionally, 30–50% of these patients continue to experience seizures even after surgical treatment [5]. Therefore, understanding the mechanisms underlying epilepsy is crucial, as it holds great potential for developing new treatment strategies.

**Fig. 1:**
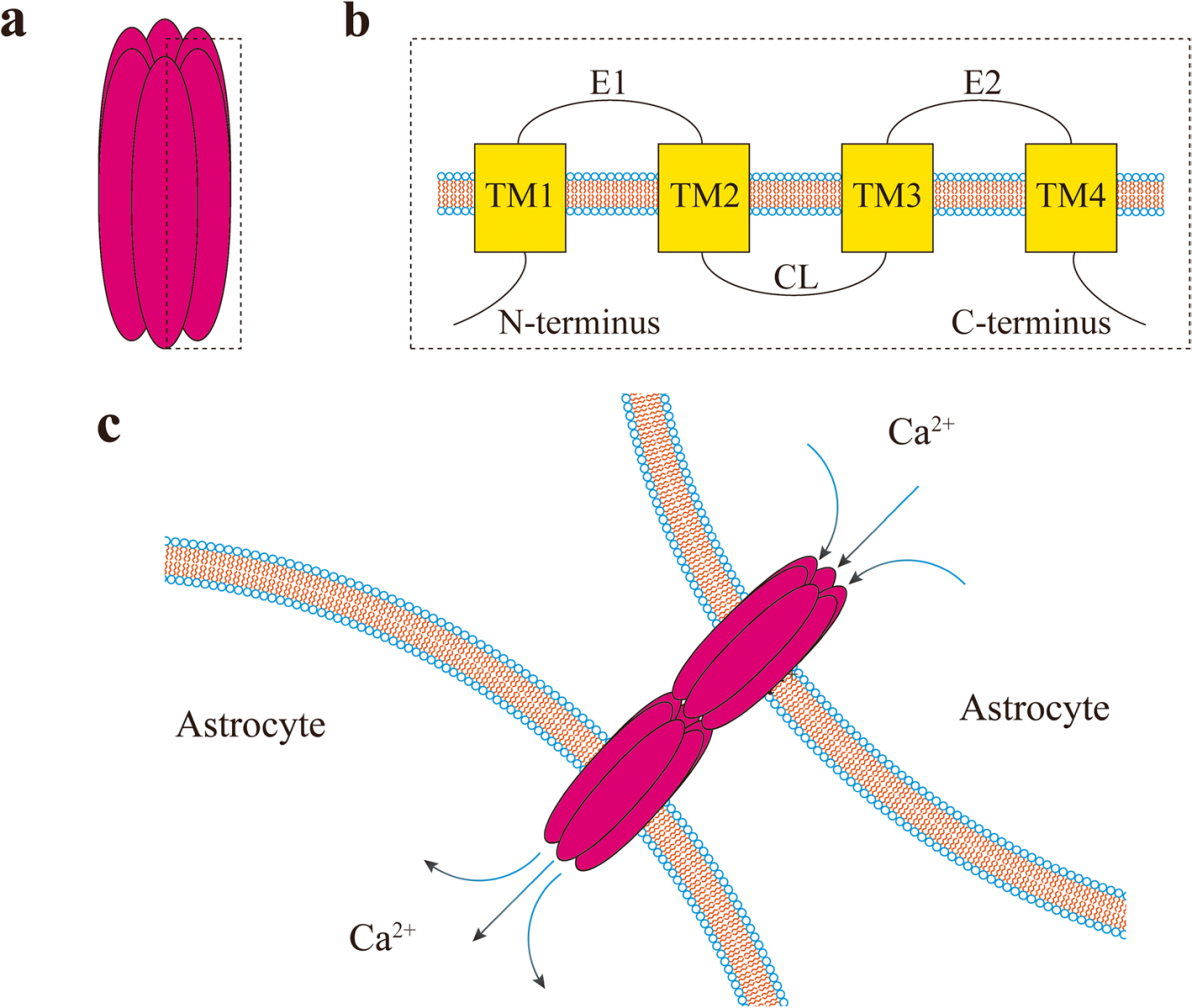
The structure of connexins, connexin-based hemichannel and connexin-based gap junctions. This figure showed the structure of connexins, connexin-based hemichannel and connexin-based gap junctions. **a** Connexin-based hemichannel: the hemichannel is formed by six connexins (one connexin drawn in the dotted line) arranged side by side, forming a channel that can only accommodate molecules of 1 kDa. **b** Connexin tetrasaccharide transmembrane (TM) domain protein: TM1 and TM2 or TM3 and TM4 are connected by extracellular loops (E1 or E2, respectively). TM2 and TM3 are connected by cellular loops (CL). TM1 ligates the N-terminus of the entire connexin intracellularly, and TM4 ligates the C-terminus of the entire connexin intracellularly. **c** The process of calcium transfer between astrocytes through Cx43-based gap junctions. After an extensive influx of calcium, the concentration of calcium in astrocytes increases, which can facilitate cell-to-cell diffusion through Cx43-based gap junctions

A connexon, formed by the binding of six connexins, can undergo extracellular glycosylation to form a hemichannel. Two connexons can couple together to form a gap junction (GJ) protein [6]. Hemichannels are formed through extracellular glycosylation of connexons. Each connexin protein subunit consists of four tetraspan transmembrane (TM) domains with intracellular N-termini and C-termini [7, 8] (Fig. 1a, b). Astrocytes can regulate synaptic transmission homeostasis through GJ-mediated release of glial transmitters such as glutamate, ATP, and D-serine, thus influencing the pathophysiology of epilepsy [9, 10]. Among the gap junctions, connexin 43 (Cx43) -based GJs are the most abundant in astrocytes. Astrocytes are extensively coupled with each other through large amounts of Cx43 [11].

GJs are essential channels for mutual coupling between astrocytes. Hemichannels composed of six connexins allow the passage of small molecules with a mass of less than 1 kDa, such as potassium, calcium, ATP, and glutamate [12]. Through the regulation of different mechanisms, GJs can promote the distribution of glucose and lactate on astrocytes [13], maintain the membrane potential and potassium equilibrium of neurons and glial cells in conjunction with Kir4.1 and Aquaporin 4 [14–16], provide spatial buffering of calcium and glutamate [17, 18], maintain syncytial isopotential by continuously balancing the membrane potential of astrocytes through electrical coupling [19], and release ATP that can activate purinergic receptors on cells [20].

The pathogenesis of epilepsy is highly complex. In recent years, a growing number of studies have focused on astrocyte involvement in epilepsy [21–23]. Among these, the role of astrocyte junction proteins in the mechanism of epileptic seizures has emerged as a research hotspot. Many scholars suggest that GJs could serve as potential targets for epilepsy treatment [11, 24–27].

## The mechanism of connexin involvement in epilepsy

### GJ and calcium ions

Astrocytes, extensively connected by GJs, form a network-like structure [28]. Thus, the excitation of astrocytes can lead to widespread membrane potential changes. Cx43-expressing astrocytes with altered potentials induce extensive synchronized calcium influx, known as calcium waves or calcium events, which are highly temporally correlated with increases in intracellular calcium concentration [29]. Calcium waves can propagate calcium ions through GJs or activate inflammatory molecules to transmit excitatory signals to distal astrocytes [30, 31]. This, in turn, causes significant changes in astrocyte membrane potential, leading to abnormal discharges that can trigger epileptic seizures. Locally unsynchronized changes in calcium concentrations caused by calcium waves may inhibit local astrocyte excitation, while widely synchronized changes can promote it [32] (Fig. 1c).

### GJ and inflammatory molecules

**Fig. 2:**
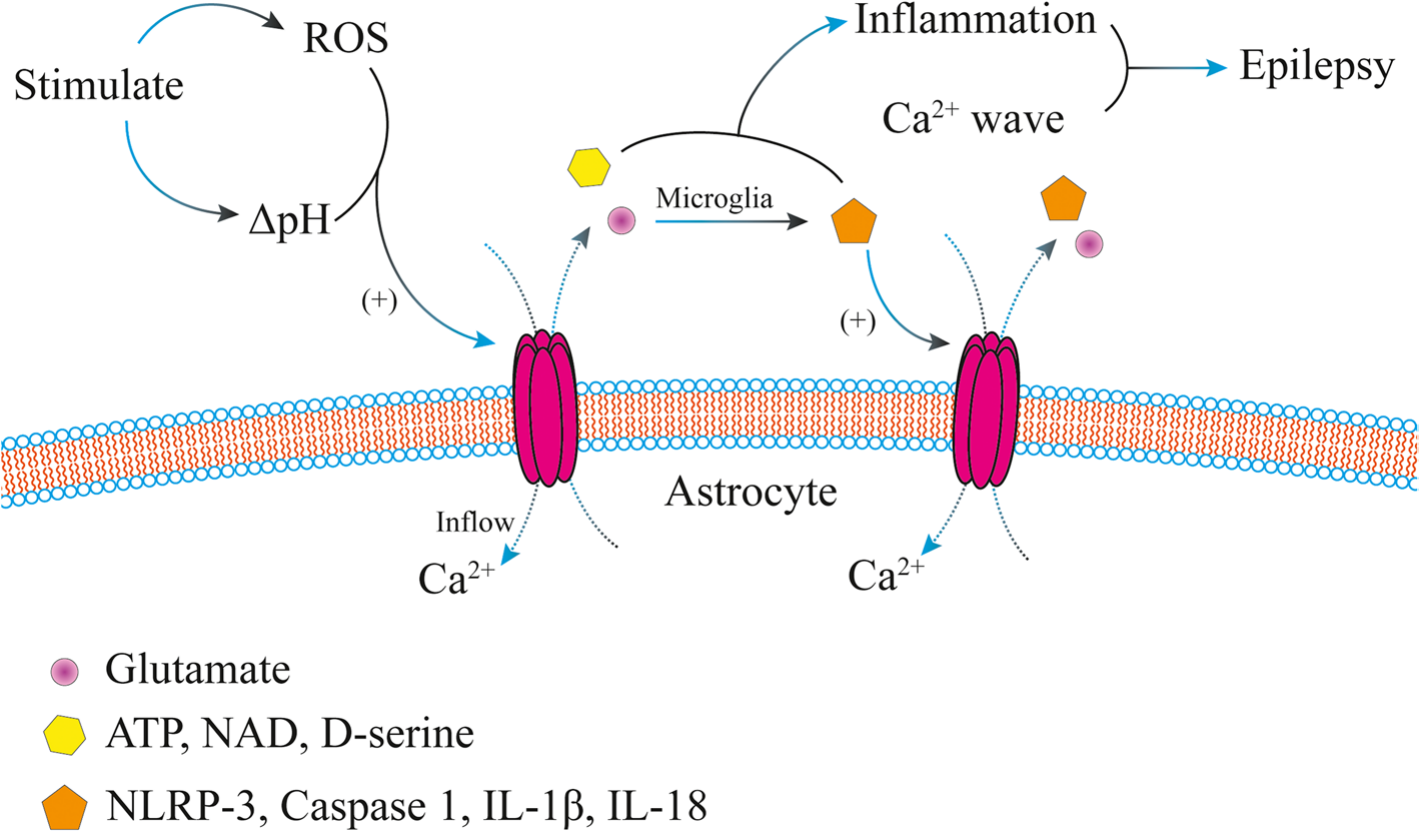
The epileptogenic mechanism of Cx43. This figure showed the epileptogenic mechanism of Cx43. Cx43-based hemichannels are involved during seizures. After receiving external stimuli, the body can activate a Cx43-based hemichannel by changing the pH in the body or producing reactive oxygen species (ROS), through which calcium then flows inward. A wide influx of calcium can form a whole wave of calcium and participate in the formation of seizures. In addition, Cx43-based hemichannels can release substances such as glutamate, adenosine triphosphate (ATP), nicotinamide adenine dinucleotide (NAD), and D-serine upon activation. Among them, ATP, NAD and D-serine can participate in the inflammatory response of the central nervous system and then participate in the formation of seizures. Glutamate activates astrocytes or microglia expressing NOD-like receptor thermal protein domain associated protein 3 (NLPR-3) and cysteinyl aspartate specific protease 1 (caspase 1), releases substances such as interleukin-1β (IL-1β) and interleukin-18 (IL-18), participates in the inflammatory response and seizure formation of the central nervous system, and activates other Cx43-based hemichannels, which in turn form a wide calcium influx to form calcium waves

The onset of epilepsy is highly correlated with the inflammatory response of the central nervous system [33], and GJs and hemichannels composed of connexins can be activated by inflammatory molecules. Different types of stimuli can cause changes in intracellular pH to open GJs and hemichannels formed by connexins [34], resulting in glutamate, D-serine, nicotinamide adenine dinucleotide (NAD), and ATP being released through hemichannels or transmitted between astrocytes through the GJ. Elevated glutamate concentrations cause microglia or astrocytes to express nucleotide-binding and oligomerization domain-like receptor thermal protein domain associated protein 3 and cysteinyl aspartate specific protease 1 (caspase 1). Caspase 1 further forms interleukin-1β (IL-1β) and interleukin-18 (IL-18). IL-1β and IL-18 are released extracellularly, which in turn activates GJs and hemichannels to cause seizures [35]. Reactive oxygen species generated during seizures also promote the opening of GJs and hemichannels [36]. In neurons, the openings of GJs and hemichannels increase abnormal hyperexcitability and synchrony, thereby strengthening the cycle of seizures [37] (Fig. 2). In conclusion, Cx43 is an important signaling channel between astrocytes and is involved in seizures and neuroimmune responses. Therefore, Cx43 may be a potential target for activating astrocyte activity [38].

### GJ and Wnt/β-catenin pathway

**Fig. 3:**
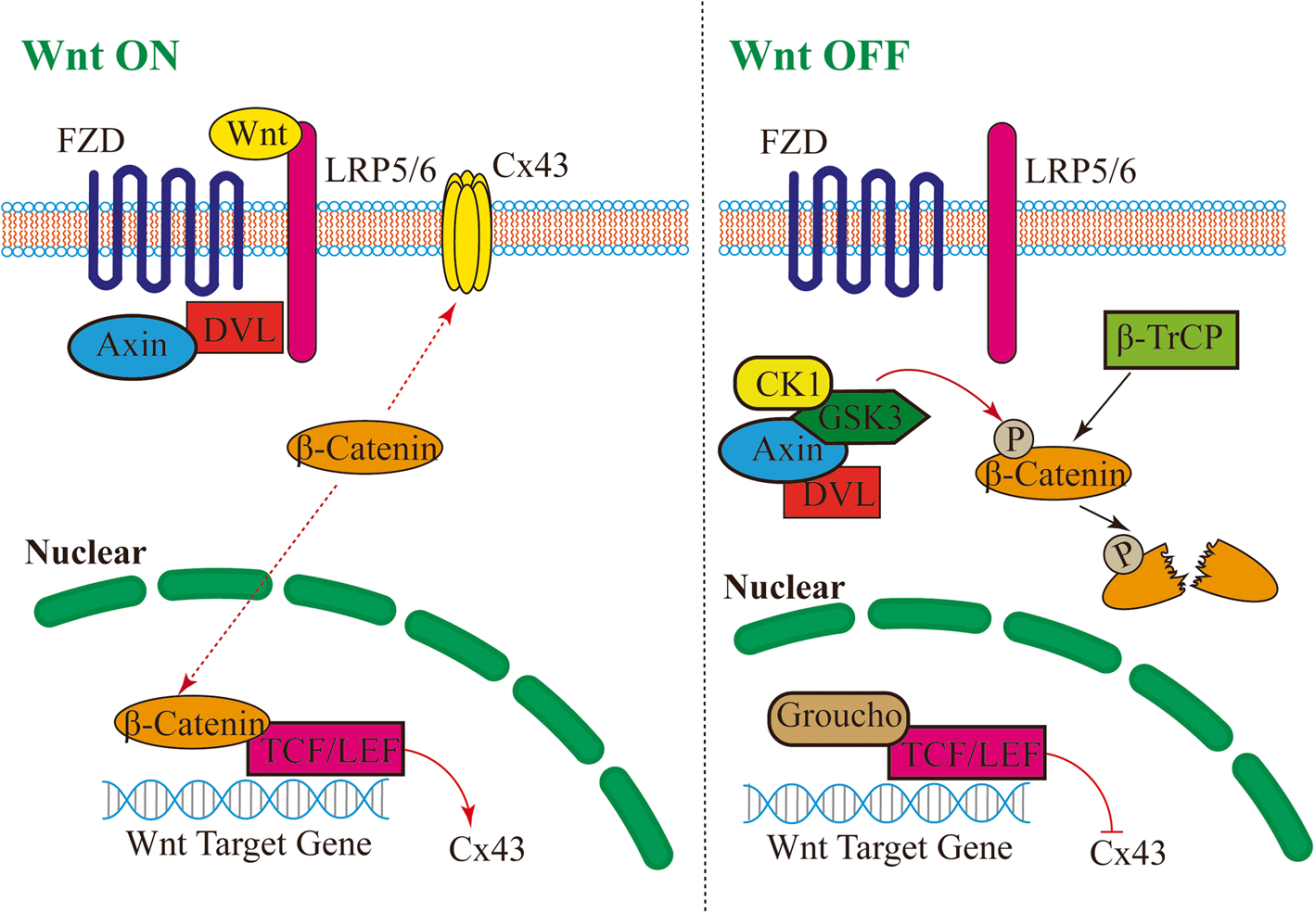
The expression of Cx43 is regulated by the canonical Wnt/β-catenin pathway. This figure showed the expression of Cx43 is regulated by the canonical Wnt/β-catenin pathway. The expression of Cx43 is regulated by the canonical Wnt/β-catenin pathway. In the "OFF" state of the canonical Wnt/β-catenin pathway (lack of Wnt), β-catenin in the cytoplasm is selectively degraded by two multistructural domain scaffold protein axis inhibitor proteins (Axin) and Adenomatous Polyposis Coli gene protein (APC), which are selectively degraded by glycogen synthase kinase-3 (GSK3) and casein kinase 1 (CK1) to promote the amino-terminal phosphorylation of β-catenin. Phosphorylated β-catenin is recognized by the β-transducin repeat-containing protein (β-Trcp), which in turn is degraded. In the "ON" state of the Wnt/β-catenin pathway (presence of Wnt), Wnt forms a trimer with Frizzled (FZD) and Lipoprotein Receptor-related Protein 5 or 6 (LRP5/6). This trimer stabilizes β-catenin by aggregating much Dishevelled (DVL) and Axin in the intracellular region of FZD and LRP5/6, which inhibits phosphorylation of β-catenin. Elevated levels of β-catenin promoted the interaction of the β-linked protein with T-cell factor/lymphoid enhancer-binding factor (TCF/LEF) transcription factors, which activated the expression of the *Cx43* gene

The canonical Wnt/β-catenin pathway is another factor that regulates the expression of Cx43. When the Wnt/β-catenin pathway is inactive (lack of Wnt), two multistructural domain scaffolding proteins, axis inhibitor (Axin) and adenomatous polyposis coli (APC), selectively degrade β-catenin in the cytoplasm. These proteins promote the amino-terminal phosphorylation of β-catenin via glycogen synthase kinase-3, leading to a decrease in β-catenin concentration outside the nuclear membrane due to the degradation of phosphorylated β-catenin by the E3 ubiquitin ligase β-transducin repeat-containing protein [39]. When the Wnt/β-catenin pathway is active (Wnt is present), Wnt binds with Frizzled and LRP5/6 to form a trimer. The intracellular regions of Frizzled and LRP5/6 then aggregate large amounts of dishevelled and Axin, thereby inhibiting β-catenin phosphorylation and degradation [40]. The increased levels of β-catenin in the cytoplasm and nucleus facilitate its interaction with TCF/LEF transcription factors, subsequently stimulating the expression of the *GJA1* gene [41] (Fig. 3). The involvement of the canonical Wnt/β-catenin pathway in epileptic seizures has been confirmed [42, 43]. Therefore, this pathway may participate in the electrical signal transduction of epileptic seizures by increasing the expression of Cx43, although further research is needed to confirm this hypothesis.

## Connexin 43 involvement in different kinds of epilepsy

Previous studies demonstrated that carbenoxolone and mefloquine significantly attenuate seizures. Both agents can block GJ, hemichannels, and pannexin 1 (Panx1). Conversely, the GJ coupling agent trimethylamine significantly enhanced seizures, indicating a strong association between connexins and epilepsy and highlighting the need for further investigation [44–48].

### Temporal lobe epilepsy with hippocampal sclerosis

Temporal lobe epilepsy (TLE) is a focal epilepsy commonly localized in the hippocampus, where irreversible sclerotic changes often occur. Many TLE patients continue to experience seizures even after anti-seizure medication therapy [49]. Bedner et al. [21] examined the hippocampi of patients with mesial TLE, including both sclerotic and non-sclerotic cases, as well as hippocampal specimens from mice with TLE. They found that patients with mesial TLE with hippocampal sclerosis and mice with mesial TLE exhibited atypical astrocyte current patterns (input resistance > 40 MΩ and/or voltage- and time-dependent currents). This indicated a lack of GJ coupling between astrocytes in the specimens. In contrast, non-sclerotic specimens showed many astrocytes with GJ coupling, suggesting that GJ decoupling is a crucial step in the pathogenesis of TLE with hippocampal sclerosis.

Vincze et al. [50] investigated the effects of modulating GJs in hippocampal slices from mice. When the GJ coupling agent trimethylamine was applied, epileptic seizures were enhanced in 12 hippocampal slices treated with low magnesium-ion artificial cerebrospinal fluid. Conversely, only two of 12 hippocampal slices treated with carbenoxolone, a specific GJ blocker, exhibited seizure-like events, and the duration of status epilepticus was shorter compared to untreated controls. When carbenoxolone was applied to trimethylamine-treated hippocampal slices, it inhibited seizure-like events in 11 out of 12 slices. Additionally, using an antibody targeting the gating peptide segment of connexin 43 (Cx43) in trimethylamine-treated slices completely eliminated seizure-like events in 5 out of 5 slices. However, blocking connexin 36 (Cx36) with quinine did not stop seizure-like events, which persisted in 7 out of 11 slices. These findings suggest that Cx43 plays a more significant role in seizure occurrence than other connexins.

Furthermore, Guo et al. [51] treated TLE mice with D4 ([R]−2-[4-chlorophenyl]−2-oxo-1-phenylethyl quinoline-2-carboxylate), a compound that inhibits the expression of Cx43-based hemichannels. They observed that pilocarpine treatment significantly up-regulated the area of glial fibrillary acidic protein (GFAP) reactivity in all regions, particularly in the hippocampus, indicating increased astrocyte activation. Pre-administration of D4 reduced astrocyte activation, as shown by a decreased GFAP-covered area in the anterior piriform cortex but not in the hippocampus. This suggests that D4-treated TLE mice effectively reduce neuroinflammation and prolong astrocyte and microglia activation compared to controls. Post-epileptic treatment with D4 reversed glial activation, normalized neuroinflammation, and restored mRNA levels of neuroinflammatory and synaptic genes. These results highlight the critical role of connexin-based hemichannels in the onset of TLE, potentially influencing future anti-seizure medication development.

### Hypothalamic hamartoma-related epilepsy

Hypothalamic hamartoma (HH) is a rare congenital malformation of the ventral hypothalamus, typically located posterior to the pituitary stalk. Approximately 40% of patients with HH suffer from refractory epilepsy [52, 53]. In vivo experiments have shown that the levels of Cx43 in HH tissues are significantly higher compared to normal human hypothalamic control tissues [27]. The researchers used mefloquine to antagonize the GJ channel in HH tissue slices and found that abnormal discharges significantly decreased both during seizures and in the interictal period. Additionally, the action potential discharge of small GABAergic neurons was affected, suggesting that GJs are involved in epileptiform discharges in HH. However, the specific epileptogenic mechanism of GJs in HH tissues requires further investigation.

### Tuberous sclerosis complex-related epilepsy

Tuberous sclerosis complex (TSC) is an autosomal disorder in which epilepsy is the most common neurological symptom, affecting approximately 80–90% of cases [54]. Xu et al. [55] found that in mice with *TSC1* gene knockout, Cx43 was expressed at lower levels in astrocytes compared to wild-type mice. These TSC1-deficient mice also exhibited abnormally elevated extracellular potassium ion concentrations following stimulation, a phenomenon similar to that seen in wild-type mice treated with carbenoxolone. Moreover, rapamycin was able to reverse both the low expression of Cx43 and the elevated extracellular potassium concentrations in TSC1-deficient mice, suggesting that the mTOR pathway may mediate the mechanism of connexin activation in TSC.

### Glioma-related epilepsy

Gliomas are malignant tumors characterized by diffuse invasive growth [56]. Epilepsy is a common symptom associated with gliomas, with an incidence of 70–90% in low-grade gliomas and 30–62% in high-grade gliomas. This difference is due to the slower growth and increased isocitrate dehydrogenase 1/2 mutations in low-grade gliomas [57]. Cx43 is primarily found in astrocytes surrounding gliomas [58]. The role of Cx43 in gliomas is complex; it can inhibit tumor growth and proliferation. Current research indicates that the use of toluidine, selective β2-AR agonists, 17β-estradiol, ciliary neurotrophic factors, and low-dose gamma radiation can regulate Cx43 expression as a potential treatment strategy for glioma [38, 59].

However, there is no clear evidence of a significant association between Cx43 and glioma-related epilepsy. Seizures are a common symptom of glioma [57]. Therefore, we believe that there is an association between Cx43 and glioma-related epilepsy, but there is a lack of research in this area [60].

## Other connexins involvement in epilepsy

Other connexins are also closely associated with epilepsy. And Cx36, connexin 32 (Cx32), connexin 30 (Cx30) and Panx1 have received increasing attention. The structure and function of these connexins are similar to Cx43, but there are still some differences. First of all, the molecular weight of Cx43 is not the same as that of other connexins. Cx43 has a molecular weight of 43 kDa, while other connexins have corresponding molecular weights. Second, the predominantly distributed cells of Cx43 are different from some other connexins. Cx43 is only distributed in astrocytes in the central nervous system, while some other connexins can be distributed in neurons. Third, the mechanism of astrocytes involved in epilepsy as described in this review applies only to connexin which is expressed in astrocytes and not in neurons. Some of the other connexins are not only involved in neuroimmune mechanisms and Wnt pathways in this review, but also through neurons.

### Connexin 36

Cx36, encoded by the *GJD2* gene, has a predicted molecular mass of 36 kDa in both mice and humans and is often distributed in neurons and microglia [61]. In previous studies, changes in Cx36 have been found in some epilepsy models. Wu et al*.* [62] detected Cx36 in the hippocampus of mice at 1 h, 4 h, one week, and two months after pilocarpine-induced epilepsy and found a large amount of Cx36 expression in each assay. Brunal et al*.* [63] used a pentylenetetrazol-induced epilepsy model in zebrafish and found that zebrafish with deletion of the *GJD2* gene were more resistant to pentylenetetrazol than wild-type zebrafish. Activating metabotropic glutamate receptors can increase Cx36 expression in neurons in a mouse model with 4-aminopyridine-induced epilepsy, while the expression of Cx36 can be effectively reduced by inactivating metabotropic glutamate receptors, which suggests a correlation between Cx36 and the glial transmitter glutamate during epileptic seizures [64].

### Connexin 32

Cx32 is encoded by the *GJB1* gene and is primarily expressed in neurons and oligodendrocytes in both mice and humans [8]. Addis et al. [65] reported two cases of *GJB1* gene copy number variation in children with mesial TLE. Increased transcription of GJB1 mRNA has also been observed in a 4-aminopyridine-induced epilepsy model in Wistar rats [66]. However, these studies only indicate an increase in *GJB1* mRNA transcription in patients or animal models with epilepsy. The role of *GJB1* mRNA during epileptic seizures and the involvement of Cx32 in epileptic processes have yet to be confirmed. Akbarpour et al. [67] implanted stimulation and recording electrodes in the right amygdala of rats and stimulated them daily at the after-discharge threshold. No increase in *GJB1* mRNA transcription was observed in the hippocampus during the initial and mid-term stages of focal seizures in the kindling model. However, *GJB1* mRNA transcription decreased during the end stage with generalized seizures. Thus, the association of Cx32 with the pathogenesis of epileptic seizures remains unclear.

### Connexin 30

Cx30 is encoded by the *GJB6* gene and is primarily expressed in neurons and microglia in both mice and humans [68]. Akbarpour et al. [67] reported that the astrocytic network of Cx30, located in perivascular regions of the hippocampus, is overexpressed at the onset of kindling to help clear excitotoxic molecules from the environment in kindling epileptogenic models. Andrioli et al. [69] induced status epilepticus in mice using pilocarpine and found that the mRNA expression of Cx30 progressively decreased. Additionally, 24 h after the epileptic seizure, the expression of Cx30 significantly decreased in the neocortex, hippocampus, and thalamic regions.

### Pannexin 1

Panx1 is a crucial channel for ATP conduction in astrocytes and neurons. Although it belongs to a different protein family than the various connexins mentioned earlier, it shares a similar topology. Initially, Panx1 was thought to form GJs on astrocytes; however, it was later shown that Panx1 is glycosylated outside the cell membrane and can only form hemichannels. Despite this, its function is closely related to GJs and can be inhibited by carbenoxolone [70, 71]. The expression of Panx1 channels is increased in a mouse model of kainic acid-induced epilepsy [72], but whether Panx1 mainly activates or inhibits epilepsy remains controversial. In epilepsy animal models, both Panx1 channel blockers and *Panx1* gene knockout can effectively suppress seizures [73, 74]. Scemes et al. [72] discovered that the loss of Panx1 in astrocytes enhanced seizure attacks in a kainic acid-induced acute epilepsy model, while the loss of Panx1 in neurons attenuated seizure manifestations, suggesting that Panx1 has a complex epileptogenic mechanism. Currently, Panx1 is generally believed to contribute to epileptic seizures by releasing the excitatory transmitter ATP in response to various stimuli (e.g., mechanical stimulation, hypoxia, increased extracellular potassium and calcium concentrations), which initiates a positive feedback mechanism in epilepsy [75]. Inhibiting Panx1 in astrocytes results in the release of high mobility group box-1 protein, indicating that Panx1 may be involved in central nervous system inflammation and seizure development [76]. Aquilino et al. [74] found that seizure activity was suppressed in Panx1 knockouts and by applying the Panx1 channel blocker Brilliant Blue-FCF in pentylenetetrazol-induced seizures. In response to pentylenetetrazol, wild-type mice experienced severe seizures for a greater proportion of time compared to Panx1-deficient mice. These findings suggest that the specific blockade of Panx1 could provide new insights for developing novel anti-seizure medications.

**Fig. 4:**
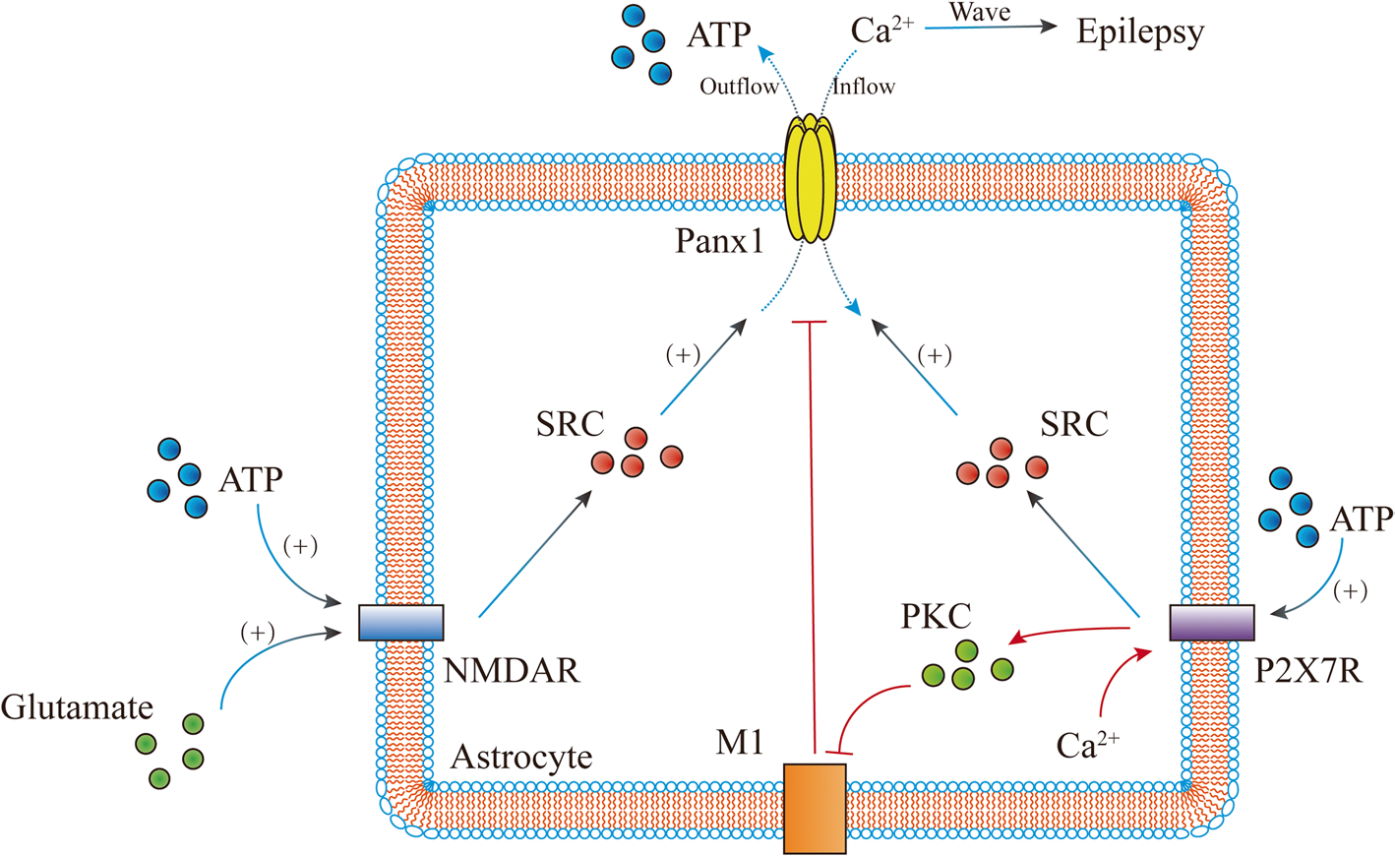
The epileptogenic mechanism of Panx1. This figure showed the epileptogenic mechanism of Panx1. Panx1-based hemichannels are involved in epileptic seizures. After the body is stimulated, according to the above mechanism, astrocytes produce a large amount of the glial transmitter glutamate, which can activate astrocytes’ N-methyl-D-aspartate receptor (NMDAR) to generate SRC to activate Panx1-based hemichannels, and calcium ions pass through this channel. The flow participates in the formation of calcium ion waves and releases adenosine triphosphate (ATP) through this channel. The released ATP can activate other NMDARs again and activate P2X7R to generate SRC, and the subsequent mechanism is the same as that of NMDAR. In addition, a large influx of calcium in astrocytes can act on P2X7R to generate protein kinase C (PKC), which inhibits the effect of muscarinic receptor 1 (M1), thereby inhibiting the effect of Panx1-based hemichannels and inhibiting epileptic seizures

Src, a tyrosine kinase, activates the Panx1 channel. When the N-methyl-D-aspartate (NMDA) receptor is activated by glutamate, Src is released into the cell and acts on the Panx1 channel, which subsequently releases ATP to surrounding or downstream neurons [77, 78]. Additionally, ATP released by astrocytes can act on the NMDA receptor to facilitate the release of intracellular Src [79]. The purinergic P2X7 receptor can also bind to ATP released by Panx1, triggering the release of Src, activating or enhancing Panx1 activity, and inducing seizures [80, 81]. Furthermore, intracellular calcium ions can act on P2X7 receptors to inhibit muscarinic class 1 receptors by releasing protein kinase C into cells, thereby closing or inhibiting Panx1 channels and suppressing seizures [20, 82] (Fig. 4).

## Conclusions

Previous studies have shown that GJs and hemichannels formed by connexins in astrocytes are involved in mediating seizures through calcium ion waves and the inflammatory response of the nervous system. The application of compounds acting on GJs or hemichannels can significantly inhibit seizures. Cx43 and Panx1 have become a research hotspot of hemichannels in recent years, but its epileptogenic mechanism is not relatively clear. Additionally, the role of connexins in the process of epilepsy is not completely clear. The use of Cx43 or Panx1 blocker Carbenoxolone has a significant inhibitory effect on seizures [45]. However, based on the involvement of Cx43 and Panx1 in cardiac electrical activity and embryonic development [83, 84], the use of Carbenoxolone as an anti-seizure medications may still need to be explored.

This review introduces the neuroimmune mechanism and Wnt pathway mechanism of Cx43 involved in epileptic seizures, and the mechanism of Panx1 involved in epileptic seizures. It is expected that research on connexins in the pathogenesis of epilepsy will be more systematic and comprehensive in the future, providing a new research direction and theoretical basis for the development of antiseizure medications and related targeted medications.

## Data Availability

Not applicable.

## Abbreviations

Caspase 1: Cysteinyl aspartate specific protease 1
Cx: Connexin
FCD: Focal cortical dysplasia
GFAP: Glial fibrillary acidic protein
GJ: Gap junction
HH: Hypothalamic hamartoma
Panx: Pannexin
TLE: Temporal lobe epilepsy
TM: Transmembrane
TSC: Tuberous sclerosis complex
